# ESR Essentials: juvenile idiopathic arthritis; what every radiologist needs to know—practice recommendations by the European Society of Paediatric Radiology

**DOI:** 10.1007/s00330-025-11891-9

**Published:** 2025-08-19

**Authors:** Sílvia Costa Dias, Celine Habre, Pier Luigi Di Paolo, Paola d’Angelo, Thomas A. Augdal, Oskar W. Angenete, Damjana Kljucevsek, Emilio J. Inarejos Clemente, Laura Tanturri de Horatio, Karen Rosendahl

**Affiliations:** 1 Department of Medicine, Faculty of Medicine of the University of Porto (FMUP), Porto, Portugal; 2 Department of Radiology, University Hospital Center of São João Porto (CHUSJ), Porto, Portugal; 3 Pediatric Radiology Unit, Radiology Division, Diagnostic Department, University Hospitals of Geneva, Geneva, Switzerland; 4 Department of Imaging, IRCCS Bambino Gesù Children’s Hospital, Rome, Italy; 5 Department of Clinical Medicine, UiT the Artic University of Norway, Tromsø, Norway; 6 Department of Radiology, University Hospital of North Norway, Tromsø, Norway; 7 Department of Radiology and Nuclear Medicine, St. Olav University Hospital, Trondheim, Norway; 8 Faculty of Medicine and Health Sciences, Institute for Circulation and Medical Imaging, Norwegian University of Science and Technology, Trondheim, Norway; 9 Department of Radiology, University Children’s Hospital Ljubljana, Ljubljana, Slovenia; 10 Department of Diagnostic Imaging. Sant Joan de Deu Barcelona Children Hospital, University of Barcelona, Barcelona, Spain

**Keywords:** Child, Arthritis, (Juvenile), Diagnostic imaging, Ultrasonography, Magnetic resonance imaging

## Abstract

**Abstract:**

Juvenile Idiopathic Arthritis (JIA) is a major contributor to chronic diseases, affecting around 1–2 in 1000 children under the age of 16. With modern treatments, the morbidity has been reduced; however, there is increasing evidence that many, if not most, children with JIA will have a chronic disease with ongoing activity into adulthood. Many studies discuss the possibility of an early window of opportunity in which patients have the best chance of responding to therapy, thereby underscoring the importance of timely and appropriate imaging. Children typically present at 4–5 years of age with one or more stiff and painful joints. If JIA is suspected, the child should undergo an ultrasound of the involved joint(s), performed by a radiologist with experience in paediatric imaging. If this is normal, with no abnormal laboratory tests and low clinical suspicion of JIA, no further imaging is required. If there is inconsistency between ultrasound and clinical findings, then they should proceed to MRI, including intravenous contrast, of the involved joint. Additional radiographs, or low-dose CT for the axial joints to examine for potential destructive change, deformation, or growth abnormalities, should be considered. In children presenting with monoarthritis, bacterial infection must be ruled out.

**Key Points:**

- *Ultrasound is the initial modality in the diagnosis of JIA, and if there is inconsistency between ultrasound and clinical findings, MRI should be performed*.
- *Radiography for the assessment of destructive change, deformity, and malalignment should be considered, alternatively, low-dose CT for the temporomandibular and sacroiliac joints and the cervical spine*.
- *Knowledge of normal imaging features in children is mandatory*.

## Key recommendations

- In the clinical setting of suspected JIA, the appropriate initial imaging protocol is a high-resolution ultrasound examination of the involved joint(s) and tendons, including Doppler. In case of monoarthritis, bacterial arthritis should be excluded. If ultrasound is inconclusive, an additional MRI with contrast should be performed. If a diagnosis of JIA is reached, an MRI of the temporomandibular joints (TMJ) should be contemplated, as involvement of these joints is asymptomatic in a high proportion of patients (level of evidence: moderate).
- For assessment of destructive change, deformation or growth abnormalities, radiography, or low-dose CT for the axial joints and the TMJs, is recommended (level of evidence: moderate).
- Knowledge on normal references for the amount of joint fluid and synovial thickness on ultrasound and MRI, as well as on bone marrow findings on MRI, is crucial to reduce misdiagnosis (level of evidence: moderate).

## Introduction

Juvenile idiopathic arthritis (JIA) is a major contributor to chronic disease in childhood, affecting around 1–2 in 1000 children (f:m 3:1) under the age of 16 [1]. It is characterised by chronic synovial inflammation, with a potential risk of developing progressive joint destruction and serious functional disability. The aetiology is unknown [2]. Historically, the disease was characterised by high morbidity, such as physical disability and loss of mobility due to joint contractures and destruction from long-standing inflammation, pain, and fatigue. In addition, drugs like glucocorticoids add to morbidity by side effects such as growth and metabolic disturbances (osteoporosis). With modern treatment such as Methotrexate, introduced during the 80s and the biologics, introduced in the late 90s, the morbidity has been reduced, but still children are faced with periods of active disease with pain and reduced mobility, and the observed side effects of the new drugs are concerning [3, 4]. Moreover, there is increasing evidence that many, if not most, children with JIA will have a chronic disease with ongoing activity into adulthood [5, 6], thus underscoring the importance of early diagnosis and instigation of appropriate treatment.

## Diagnosis and the role of imaging

JIA is a heterogeneous disease, classified into seven distinct categories: oligoarthritis, polyarthritis, systemic arthritis, psoriatic arthritis, enthesitis-related arthritis, and undifferentiated arthritis, of which oligoarthritis is the most common [7]. Although the diagnostic criteria are based on history and clinical and laboratory findings, imaging has become an important tool both in narrowing the differential diagnosis and monitoring disease activity, development, and potential complications to treatment. While evaluation of disease activity is performed with ultrasound and/or magnetic resonance imaging (MRI), joint damage and secondary growth disturbances are still performed radiographically [8]. However, there is a lack of comprehensive imaging guidelines across specialities.

## Imaging techniques

### Ultrasound with Doppler

**Fig. 1:**
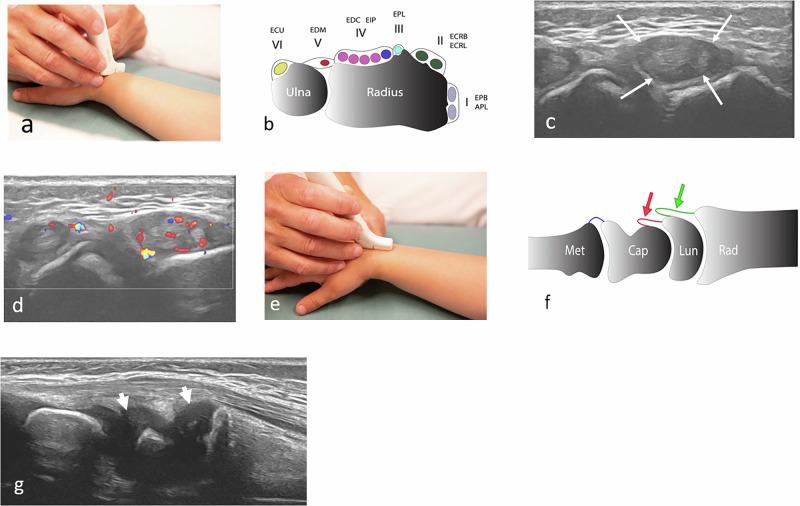
Ultrasound of the wrist in a two-year-old boy with polyarticular JIA. **a** Axial view, dorsal wrist, visualising (**b**) the extensor tendons, showing (**c**) a thickened (long arrows) and (**d**) hyperaemic synovium consistent with tenosynovitis of compartments VI and IV. **e** shows a longitudinal view through the wrist, with **f** illustrating the radiocarpal (green arrow) and midcarpal (red arrow) recesses. **g** Note the appearances of the dark cartilage, which can easily be mistaken for fluid (arrow heads). To differentiate between the two, gain might be adjusted to visualise the typical “speckles” within cartilage, as compared to the dark/black fluid

Ultrasound is a valuable tool for evaluating inflammatory change, such as joint effusion, thickened, hyperaemic synovium, tenosynovitis (Fig. 1), and enthesitis, and guiding joint injections, whilst assessment of chronic change is less reliable [9]. To differentiate between normality and pathology, knowledge of age, gender, and joint-related reference standards for the amount of joint fluid and the appearances/thickness of the synovium are required. An update on adequately sized, population-based studies reporting on ultrasound-based references is provided in Table 1. Of note is the wide variation in the amount of joint fluid seen in otherwise healthy children by age and the ultrasound technique used. Obviously, borderline findings combined with equivocal clinical findings might lead to both over and underdiagnosis of arthritis.

**Table 1:** Published reference standards for synovial thickness and joint fluid in different joints.

JOINT references	*n*	Age (years)	Cohort	Study design	Centre	*N*	Scan (sagittal)	Synovium echogenicity	Synovium thickness (bone-capsular distance, exc. knee)	Joint fluid
HIP										
Rohrschneider [47]	166	0.3–17	NA	Prospective	Mono	NA	ant rec, ER	Hypo	Mean 5.5 mm, 95% CI [5.4, 5.6]*^,#^	Presence 12%, an
Robben [48]	58	1.7–12.5	NA	Prospective	Mono	1	ant rec	Hyper/hypo	Mean 4.7 mm, 95% CI [4.5, 4.9]	Presence 4%
Tien [49]	784	2.5–6.6	(1)	NA	NA	NA	ant rec, NR	Iso	Mean 7.3 mm, 95% CI [7.2, 7.4]^#^	NA
Trauzeddel [50]	445	1–18	(2) (3)	NA	Multi	11	ant rec, NR	NA	Range 5.1–7.5 mm^**,!^	NA
Zuber [51]	408	0–18	(2)	NA	Mono	3	ant rec	NA	Med 6.7 mm, (C10-C90 5.9–7.3 mm) (≥16 y)^##,!^	Absence
Collado [52]	60	2–16	NA	NA	Mono	1	ant rec, ER	NA	Mean 5.2 mm, 95% CI [5.2, 5.2]^##^	Absence
Wittoek [53]	485	0.2–18	NA	Prospective	Mono	6	ant rec, ER	NA	Med 3.4 mm, Range (0.9–9.1)^!^	NA
KNEE										
Windshall [54]	435	1–18	NA	NA	Multi	9	suprapat	NA	Mean 3.2 mm, 95% CI [1.3–6.2] (16–18 y)^#^	Presence 64%, hypo
Collado [52]	60	2–16	NA	NA	Mono	1	suprapat	NA	Mean 1.3 mm, 95% CI [1.2, 1.4]	Presence 60%, an
Dias [55]	127	3–17	(1)	Prospective	Mono	2	suprapat	NA	Med 2.0 mm, Percentile 2.5^th^–97.5^th^ [1.4–3.3] (14–17 y)**^,^***^,##,^^!^	Presence 76%****
SHOULDER										
Trauzeddel [56]	445	1–18	(2) (3)	NA	Multi	> 3	ant rec	NA	Mean 3.5 mm, 95% CI [2.6, 4.5]**^,##^	Absence
ELBOW										
Trauzeddel [57]	437	1–18	(2) (3)	NA	Multi	> 2	ant rec	NA	Mean 3.5 mm, 95% CI [3.4, 3.6]**^,##^	NA
WRIST										
Rosendahl [58]	116	6–16	(1)	Prospective	Mono	1	dorsal rec N/F	Hypo		
RC									Mean 0.4 mm, 95% CI [0.3, 0.5]**	Presence 50%, an
MC									Mean 0.5 mm, 95% CI [0.4, 0.6]**	Presence 10%, an
FINGERS										
Collado[52]	60	2–16	NA	NA	Mono	1	dorsal rec	NA		
MCP									Mean 0.6 mm, 95% CI [0.5, 0.7]*****	Presence (some), an
PIP									Mean 0.6 mm, 95% CI [0.6, 0.7]*****	

Cohort, recruitment method: (1) population-based, (2) patients with disease not involving the joint, and (3) children of investigators/friends

Synovium thickness: the bone-capsular distance is provided with the exception of the knee, for which the thickness of the suprapatellar recess is reported

Joint fluid: percentage of joints with presence of fluid, echogenicity of fluid

*an* anechoic, *ant rec* anterior recess, *CI* confidence interval, *dorsal rec* dorsal recess, *ER* external rotation, *hyper* hyperechoic, *hypo* hypoechoic, *iso* isoechoic, *MC* midcarpal joint, *MCP* metacarpophalangeal joint, *Med* median, *Mono* monocentric, *Multi* multicentric, *n* number of included cases, *NA* not applicable, *N/F* neutral/flexion, *NR* neutral rotation, *PIP* proximal interphalangeal joint, *RC* radiocarpal joint: suprapat: suprapatellar recess, *N* total number of examiners

* For height > 100 cm; Mean 2.1 mm (< 65 cm), 5.1 mm (65–100 cm)

** Right side

*** Double synovial layer thickness with light transducer compression to squeeze the fluid away

**** In at least one of the knees

***** Second digit

^#^ Increases with age and height

^##^ Increases with age

^!^ Results could not be reported as mean and 95% CI

Several examination techniques and protocols for the assessment of joints in children with JIA have been proposed [10, 11]. The ESPR (European Society of Paediatric Radiology) has endorsed the recommendations, including a detailed description of transducers and main parameter settings, given by the ESPR musculoskeletal task force group (https://www.espr.org/app/uploads/2022-US-Joints-in-children.pdf).

As for grading of inflammatory change, several US-based scoring systems have been suggested; however, no international consensus across specialities has been reached [10]. For example, in a recent paper from the PreS (Paediatric Rheumatology European Society) Imaging Working Party, a combination of five views through the knee joint were identified as being the most sensitive for synovitis [12]. The degree of synovitis was scored in accordance with the Paediatric Outcome Measures in Rheumatology group [13], a consensus-driven, international group, defining normality as the absence of effusion without providing cut-offs between normality and pathology. Obviously, this might introduce significant overdiagnosis.

Ultrasound has a number of limitations. Firstly, the reported sensitivity for detection of both acute and chronic changes varies widely, reflecting its operator dependency and limited visualisation of the entire joint. In particular, assessment and grading of chronic change appear to be imprecise [9]. Moreover, ultrasound findings have low prognostic value in patients in remission [14], and the accuracy for detecting enthesitis has yet to be standardised and further evaluated [15].

### MRI

MRI plays an important role in assessing disease activity, as well as damage to cartilage and bone in children with JIA. However, the normal, growing skeleton often exhibits MR findings such as bone marrow oedema-like change, bony depressions, and irregular ossification; features that easily can be mistaken for true marrow oedema or erosions. For example, a study from 2008 indicated that MRI might detect erosive change of the carpals with greater sensitivity than radiography, particularly in early disease [9]. However, during normal maturation, the carpals can become quite irregular, thus mimicking pathology [16]. Moreover, bone marrow oedema might be a precursor of erosive change/joint damage progression [17, 18]. But again, since bone marrow oedema-like change, including «kissing lesions» of the carpals is a frequent finding in children, this feature must be interpreted with caution. The same goes for joint fluid, as it is difficult to differentiate when an effusion is present, given the wide variation in normal joint fluid volume (Table 1).

Nevertheless, MRI has become a useful tool for evaluating and monitoring children with JIA, particularly those with subclinical disease [18]. It is especially valuable for assessment of axial joints such as the cervical spine and sacroiliac joints (SIJ), and is also the modality of choice for assessing temporomandibular (TMJ) involvement [19].

**Fig. 2:**
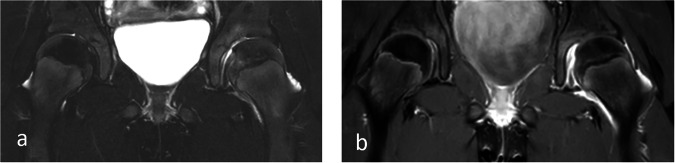
Nine-year-old girl with psoriasis-related JIA and pain in her left thigh. MRI, with (**a**) a coronal T2-weighted SPAIR sequence, showed a sliver of joint fluid in her left hip, whilst the T1-weighted, fat-saturated post-contrast image (**b**) showed vivid synovial enhancement, consistent with arthritis, underscoring the need for intravenous contrast administration to assess active inflammation

**Fig. 3:**
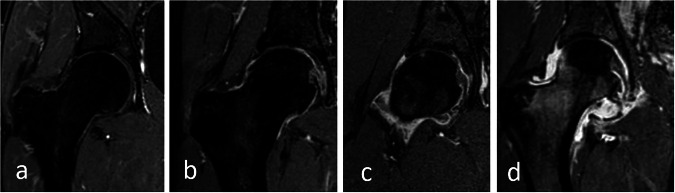
Hip MRI (T1-weighted, fat-saturated post-contrast images) in four different children aged 12 years, showing different grades of synovial enhancement: (**a**) normal, (**b**) mildly increased synovial enhancement, (**c**) moderately increased, and (**d**) severely increased synovial enhancement

Synovial inflammation, i.e. a thickened, hyperaemic synovium, most often, but not always combined with an effusion, is the key indicator of disease activity in JIA. For most joints, the use of intravenous contrast is needed to differentiate between a pathological synovial membrane and an effusion (Fig. 2). From previous work, taking the influence of timing into consideration, we know that a normal synovium is thin with barely visible enhancement [20, 21], and that there is a continuum towards a thickened, pathologiclal membrane showing vivid enhancement. To monitor disease activity, several grading systems have been established for different joints, of which the more robust ones are listed in Supplementary material A. A grading system for synovial enhancement of the hips, according to Tanturri de Horatio, is shown in Fig. 3.

Regarding the TMJ, there are suggestions for scoring systems [22, 23], but as the authors state, there are challenges regarding repeatability, also shown in a recent Dutch publication [24]. Despite its drawbacks, MRI of the TMJ is a sensitive and specific diagnostic tool in the work-up of the JIA patient.

### Technical requirements and suggested protocols

Both 1.5-T and 3-T MR machines are feasible, given the appropriate type and positioning of coils. Applying techniques to reduce the need for sedation are mandatory. However, the literature on child-specific reconstruction methods is sparse [25].

Protocols for assessing the axial joints, such as the cervical spine, sacroiliac and TMJs, and the extremities, based on previous recommendations from the ESPR-ESSR [26], as well as published updates [19, 27], are listed in Table 2. A basic protocol is suggested, together with joint–specific add-ons and potential new sequences to be considered.

**Table 2:** Suggested joint-specific MRI protocols

Basic sequences	Joints							Aims
	Knee	Ankle/foot including MTFJ	Hip	ERA − LLS + SI + hips***	Cervical spine	Wrist/hand including MCP	TMJ	
Fluid sensitive sequence*	Axial and sagittal (R), coronal (O)	Sagittal and coronal (R), axial (O)	Coronal and axial (R)	Sagittal LLS (R), coronal and axial—sacrum oriented; include hips (R)	Sagittal and axial (R), coronal (O)	Coronal and axial (R)	Sagittal oblique (R)	JE, BME, BE, cartilage loss (if PD-weighted FS used)
T1-weighted TSE	Coronal (R)	Sagittal (R)	Coronal (R)	Coronal and axial—sacrum oriented; include hips (R)	Sagittal (R)	Coronal (R)	Coronal and sagittal oblique (R)	BME, BE, growth change (TMJ)
T1-weighted FS pre-Gad	Sagittal (R)	Sagittal (R)	Coronal (R)	Coronal—sacrum oriented; includes hips (R)	Sagittal (R)	Coronal and axial (R)	Sagittal oblique (O)	Synovitis
T1-weighted FS or Dixon pos-Gad—1st acquisition 2 min.	1st Sagittal and 2nd axial (R)	1st Sagittal and 2nd coronal (R), axial (O)	1st Coronal and 2nd axial (R)	1st Coronal and 2nd axial—sacrum oriented; include hips (R)	1st Sagittal and 2nd coronal (R), axial (O)	1st Coronal and 2nd Axial (R)	Sagittal oblique for each joint and Coronal of both joints (R)	Synovitis
Additional specific sequences	Gradient echo (3D) coronal (O)	Gradient echo (3D) sagittal (O)	Gradient echo (3D) sagittal (O)	No	No	Gradient echo (3D) Coronal (O)	PD Sagittal Oblique closed & open mouth (O), T1-MPRAGE Sagittal (O)	Gradient echo for Cartilage loss and BE; PD in the TMJ for disk evaluation
New sequences to consider—FLAIR FS/DIR/qDESS	Sagittal or axial	Sagittal	Coronal	Coronal or Axial—sacrum oriented; include hips	Sagittal or axial	Coronal or axial	No	Synovitis
New sequences to consider—diffusion	Axial	Axial	Axial	Axial—sacrum oriented; includes hips	Axial	Axial	No	Synovitis
New sequences to consider—T2 Dixon**	Axial, sagittal and coronal	Sagittal and coronal; axial (O)	Coronal and axial	Sagittal LLS, coronal and axial—sacrum oriented; include hips	Sagittal, axial and coronal	Coronal and axial	Sagittal and coronal oblique	JE, BME, BE

*STIR* short tau inversion recovery, *TSE* turbo spin echo, *FS* fat suppression, *FLAIR FS* fluid attenuated inversion recovery with fat suppression, *DIR* dual inversion recovery, * qDESS* quantitative double-echo in steady-state, *R* recommended, *O* optional, *MTFJ* metatarsophalangeal joints, *MCFJ* metacarpophalangeal joints, *PD* proton density, *T1-MPRAGE* magnetisation-prepared rapid gradient echo, *JE* joint effusion, *BME* bone marrow oedema, *BE* bone erosions

* T2-weighted FS or Dixon/STIR/PD-weighted FS or Dixon

** Fat only and water only: replacing T1-weighted TS and fluid-sensitive sequences

*** Combined protocol ERA (enthesitis-related arthritis) for LLS (lower lumbar spine) + SI (sacro-iliac joints) + hips

**Fig. 4:**
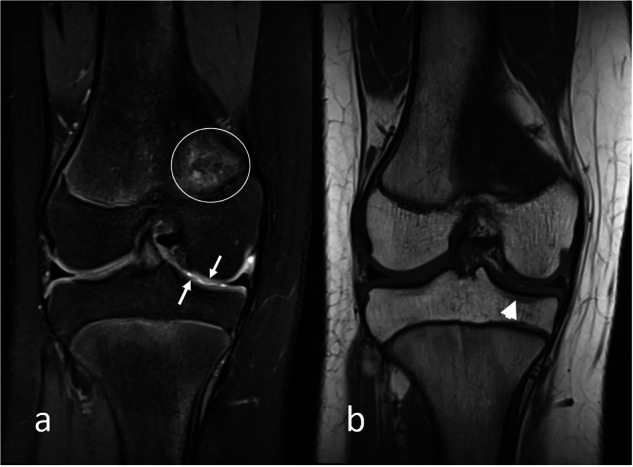
Knee MRI in an 11-year-old girl with JIA, (**a**) coronal PD-weighted fat-saturated image showing irregular cartilage in the femorotibial medial compartment (arrow) and (**b**) subtle subchondral sclerosis on a coronal T1-weighted image (arrowhead). Note the oedema in the distal medial femoral metaphysis, representing a cortical desmoid (circle)

The basic protocol should include fluid-sensitive sequences (e.g., short tau inversion recovery (STIR), T2-weighted fat-saturated (T2 FS) or T2 Dixon water only images) for the assessment of joint fluid and oedema, and a T1-weighted sequence for the assessment of growth changes and chronic structural change [19, 26]. Alternatively, a proton density (PD)-weighted FS sequence might replace the fluid-sensitive sequence for visualisation of cartilage, as well as fluid (Fig. 4). If another fluid-sensitive sequence is preferred over the PD FS sequence, an additional cartilage sequence should be considered. Finally, recent studies have shown that the T2 Dixon fat-only images can replace T1-weighted FS images, thus shortening the scan time significantly [28, 29].

As mentioned above, the use of intravenous contrast is needed to differentiate between an effusion and a hyperaemic and thickened synovial membrane. Due to diffusion of contrast into the joint fluid, the timing of post-contrast images, preferably in two planes, should be short and standardised, for instance after a 2–3 min delay [30]. See Table 2 for examples of child- and joint-specific protocols. For MR imaging of the SIJ, the role of intravenous contrast is limited, although it might be helpful in equivocal cases [26, 31].

Because the hips are commonly involved in children with juvenile spondyloarthropathy, a (true) axial acquisition with a larger field of view to visualise the entire pelvis, including the hips, has been advised [26]. This extended view also allows for assessment of pelvic enthesitis (Table 2).

### Future perspectives

During the past years, significant efforts have been made to reduce the need for intravenous gadolinium administration, both due to unwanted side effects and examination time [32]. Alternative MR sequences, such as fluid attenuated inversion recovery with fat suppression (FLAIR FS), dual inversion recovery (DIR) and quantitative double-echo in steady-state (qDESS) [33, 34] and diffusion-weighted sequences have been evaluated [35]. Currently, contrast-enhanced MRI is often indispensable in the diagnostic work-up of the JIA patient.

### Radiography

Although conventional radiography (CR) has its limitations in detecting inflammatory change, soft tissue involvement and early structural change, it still plays an important role in narrowing the differentials, particularly in children presenting with monoarthritis, and in evaluating severe joint damage and growth disturbances in children with JIA [26, 36].

**Fig. 5:**
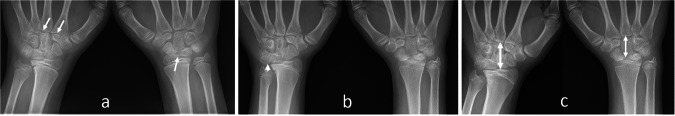
Serial radiographs of the hands in a boy with polyarticular JIA (initially HLA B 27 negative at presentation age 2 years, but positive at age 17) show the development and progression of chronic degenerative change from 12 years to 18 years of age: (**a**) active inflammation of the carpals, with osteoporosis and growth disturbances (left carpals > right), as well as structural change with joint space narrowing (left carpometacarpal, right radiocarpal (arrows), (**b**) negative left ulnar variance at age 12 years (arrowhead) and progression of right radiocarpal joint space narrowing with crowding of the proximal carpal bones, and (**c**) reduced carpal length at age 15 (double arrows), radial deviation of carpal bones and dorsal subluxation (better seen on a lateral projection)

Except for the sacroiliac joints, the TMJs and the spine [26], CR remains the preferred modality for assessment of chronic change [8], of which knees, ankles, hands and feet are the most frequent sites showing radiographic change [37]. The radiographic findings differ significantly from those in adults and vary according to mode of onset and age. In younger children, the initial findings might be more developmental rather than destructive, i.e. bony overgrowth/architectural distortion, whereas in older children and adolescents destructive changes might supervene [38] (Fig. 5). Similar, in other appendicular joints, accelerated bone maturation might result in architectural distortion and remodelling, and subsequent premature closure of the growth plate with shortening of bones. Children with later-onset JIA sometimes have destruction/erosions and narrowed joint space as the first feature, often followed by malalignment. Evaluation and grading of chronic changes can be performed by comparing serial radiographs or by using one of the scoring systems for hands [18–20] or for hips [21].

### Technical requirements and suggested protocols

High-resolution radiographs are advised. Currently, no agreed recommendations or protocols exist; however, a relatively recent systematic review [39] suggests an initial routine CR of the wrist, hands and forefeet in polyarticular JIA with positive rheumatoid factor (RF)/anti-citrullinated protein antibodies (ACPA) and in new-onset RF/ACPA-negative polyarticular JIA with adverse prognostic factors. In other JIA subtypes, routine CR is not recommended, except at the discretion of the paediatric rheumatologist for symptomatic joints. Follow-up of destructive change and/or malalignment should be guided by clinical findings. For the appendicular joints, both sides should be included to identify growth disturbances. In contrast to the adult protocol, one front view often suffices (PA of wrist/hands; AP of ankles/feet, knees, hips, shoulders, and elbows).

### Assessment of bone health

For assessment of bone health, dual-energy X-ray absorptiometry (DXA) is the most commonly used method, preferring the total body less head and lumbar spine (LS 1–4) locations [40]. To adjust for skeletal size, volume bone mineral density (or bone mineral apparent density, in g/cm^3^) is calculated, or BMD *Z*-scores are adjusted for height [41] or bone age [42]. A recent study indicates that radiogrammetry based on a hand radiograph might provide an adjuvant tool to DXA, given that thorough calibration is performed [43].

### Computed tomography (CT)

Although CT, and in particular the novel photon counting detector CT technique, provides detailed images of the osteochondral domain, the method plays a limited role in the evaluation of children with JIA. Non-contrast CT can be used to evaluate structural damage, erosions, or ankylosis, with acquisition focused on thin-section axial images and multiplanar reconstructions. Currently, it is used for assessment of joints that are difficult to examine with CR, such as the TMJ, the upper cervical spine and the sacroiliac joint [26] in cases where MRI is contraindicated. In addition, CT is useful for preoperative planning, providing high-resolution imaging that serves as a roadmap for surgical interventions such as joint replacement or arthrodesis.

### Technical requirements and suggested protocols

Modern CT scanners incorporate features that minimise radiation dose, such as iterative reconstruction algorithms, automated exposure modulation, and child-specific protocols. Key technical considerations include using multidetector CT with slice thicknesses of 0.5–1 mm for detailed imaging of small joints, employing dose-reduction techniques like low kiloVolt peak (kVp) settings (e.g. 80–100 kVp) and iterative reconstruction. Contrast is not routinely used in CT for patients with JIA due to the limited indications [44].

### CBCT of the TMJs

**Fig. 6:**
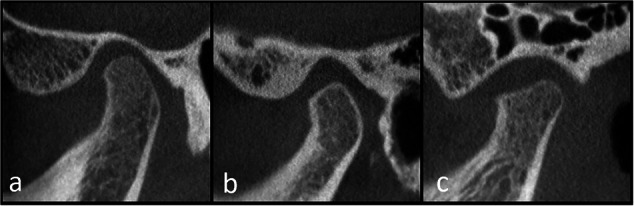
Cone-beam CT, sagittal view of the TMJ, (**a**) a 12-year-old boy with a normally shaped fossa-eminence and condyle, (**b**) a 14-year-old girl with a normal fossa, but subtle anterior flattening of the condyle, and (**c**) a 15-year-old with a moderately widened fossa-eminence and a mildly to moderately flattened condyle. This figure has been reproduced from [46] under the Creative Commons Attribution 4.0 International License

For TMJs, cone beam computed tomography (CBCT) is currently accepted as the imaging modality of choice for visualisation of bony structures [45], especially in cases where MRI is equivocal or contraindicated. It provides multiplanar reconstructions to a relatively low radiation burden as compared to conventional CT, and depicts subtle erosions, flattening of articulating surfaces, subchondral cysts, subchondral sclerosis and osteophyte formation (Fig. 6). Recently, a novel, validated scoring system for monitoring the development of chronic change was published [46].

### Differential diagnosis

In a child with acute-onset monoarthritis, the differential diagnosis must include septic arthritis, trauma, malignancy and haematological diseases, of which leukaemia is highly relevant. In addition, chronic non-bacterial osteomyelitis, postinfectious arthritis, acute rheumatic fever, and Lyme disease, among others, should be considered.

### Summary statements

**Fig. 7:**
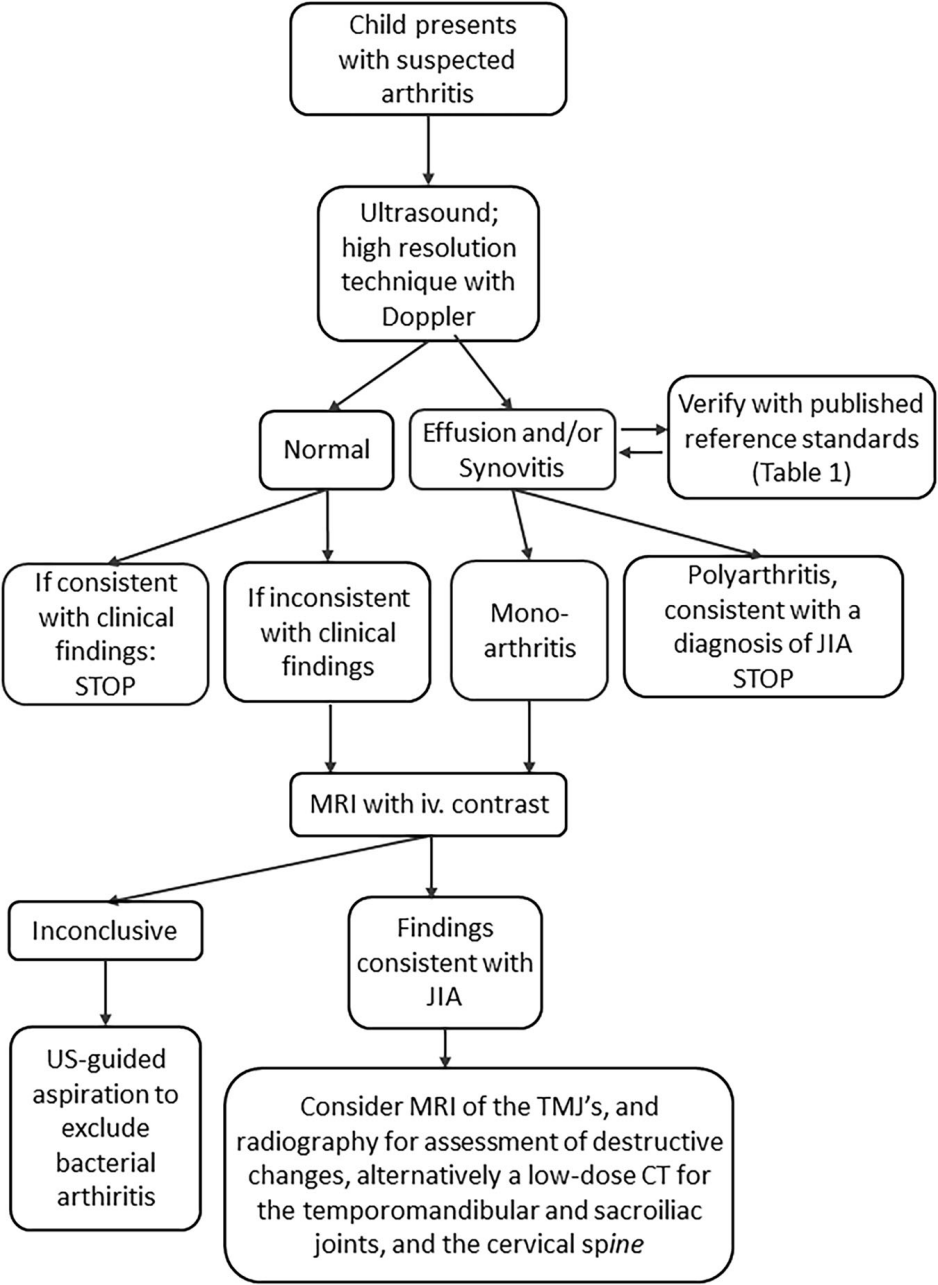
Suggested investigative imaging pathway for suspected JIA

JIA is not uncommon in clinical practice. The radiologist has an essential role in the diagnostic pathway towards an early diagnosis, to prevent chronic structural damage. An ultrasound of the involved peripheral joint(s) is a core component of the assessment and should be performed in line with the ESPR MSK task force guidelines. If inconsistency between clinical and ultrasound findings, additional imaging with MRI, including intravenous injection of a gadolinium-based contrast agent, is required. For evaluation of the axial joints, MRI is the primary diagnostic imaging tool. Plain radiographs for the assessment of destructive change, deformity, and malalignment should be considered; alternatively, a low-dose CT for the axial skeleton and the TMJs. We have provided an extensive overview of published ultrasound and MRI-based reference standards for joint fluid and synovial appearances, as well as a flow chart of imaging techniques (Fig. 7) to facilitate differentiation between normality and pathology, as well as available MR-based scoring systems for disease activity. Moreover, we have suggested feasible child- and joint-specific MRI protocols. Communication with involved clinicians is a core component of the radiological assessment.

### Patient summary

JIA is the most common rheumatological disease in childhood. This article provides an evidence-based summary of the appropriate imaging tests that children should undergo when there is clinical concern for JIA. A child-specific approach to imaging is crucial for a correct diagnosis.

## Supplementary information

ELECTRONIC SUPPLEMENTARY MATERIAL

## Abbreviations

ACPA: Anti-citrullinated protein antibodies
CR: Conventional radiography
CT: Computed tomography
DIR: Dual inversion recovery
DXA: Dual-energy X-ray absorptiometry
ESPR: European Society of Pediatric Radiology
FLAIR FS: Fluid attenuated inversion recovery with fat suppression
JIA: Juvenile idiopathic arthritis
kVp: kiloVolt peak
MRI: Magnetic resonance imaging
PD: Proton density
PreS: Pediatric Rheumatology European Society
qDESS: Quantitative double-echo in steady-state
RF: Rheumatoid factor
SIJ: Sacroiliac joints
STIR: Short tau inversion recovery
TMJ: Temporomandibular joint
