# Supplementary material for: ESR Essentials: juvenile idiopathic arthritis; what every radiologist needs to know—practice recommendations by the European Society of Paediatric Radiology
Source: Eur Radiol. 2025 Aug 19;36(2):1261–71. doi: 10.1007/s00330-025-11891-9 (PMC12953336; doi:10.1007/s00330-025-11891-9)
Supplement: Supplementary file 1 — ELECTRONIC SUPPLEMENTARY MATERIAL [file 330_2025_11891_MOESM1_ESM.pdf]

ESR Essentials: juvenile idiopathic arthritis; what every radiologist needs to know - practice recommendations by the European Society of Paediatric Radiology

Electronic Supplementary Material

Supplement A.

Validated, joint specific scoring systems for active inflammation and / or chronic change in JIA by imaging method.

**Joint**	**Radiograph**	**Ultrasound**	**MRI**	**CBCT**
**Knee**	Pettersson et al[1]	PAUSS-knee[2]<br>CARRA JIA US Workgroup[3]	JAMRIS[4]	NA
**Ankle**	NA	PAUSS-ankle[5]	NA	NA
**Hip**	Shelmerdine et al [6]<br>Bertarmino et al [7]	NA	Tanturri de Horatio et al[8]	NA
**Spine**	NA	NA	NA	NA
**SIJ**	NA	NA	SPARCC SIS: Weiss et al [9] and Panwar et al[10]	NA
**Wrist**	Ravelli et al - Adapted Sharp/van der Heijde score[11]<br>Poznanski et al [12]	NA	Malattia et al [13]<br>Damasio et al [14]	NA
**TMJ**	NA	NA	Angenete et al [15]	Augdal et al [16]

References

1. Pettersson H, Rydholm U. Radiologic classification of knee joint destruction in juvenile chronic arthritis. Pediatr Radiol. 1984;14(6):419-21. https://doi.org/10.1007/BF02343432

2. Vega-Fernandez P, Rogers K, Sproles A, Thornton S, Huggins J, Lovell DJ, Cassedy A, Meyers AB, Ting TV. Diagnostic Accuracy Study of the Pediatric-Specific Ultrasound Scoring System for the Knee Joint in Children With Juvenile Idiopathic Arthritis. Arthritis Care Res (Hoboken). 2024;76(2):251-8. https://doi.org/10.1002/acr.25218

3. Ting TV, Vega-Fernandez P, Oberle EJ, De Ranieri D, Bukulmez H, Lin C, Moser D, Barrowman NJ, Zhao Y, Benham HM, Tasan L, Thatayatikom A, Roth J, Childhood A, Rheumatology Research Alliance Juvenile Idiopathic Arthritis Ultrasound W. Novel Ultrasound Image Acquisition Protocol and Scoring System for the Pediatric Knee. Arthritis Care Res (Hoboken). 2019;71(7):977-85. https://doi.org/10.1002/acr.23746

4. Hemke R, van Rossum MA, van Veenendaal M, Terra MP, Deurloo EE, de Jonge MC, van den Berg JM, Dolman KM, Kuijpers TW, Maas M. Reliability and responsiveness of the Juvenile Arthritis MRI Scoring (JAMRIS) system for the knee. Eur Radiol. 2013;23(4):1075-83. https://doi.org/10.1007/s00330-012-2684-y

5. Vega-Fernandez P, Rogers K, Avar-Aydin PO, Quinlan-Waters M, Huggins J, Brunner HI, Lovell DJ, Altaye M, Cassedy A, Meyers AB, Ting TV. Validation of the ankle-specific pediatric arthritis ultrasound scoring system in children with juvenile idiopathic arthritis. Semin Arthritis Rheum. 2024;69:152545. https://doi.org/10.1016/j.semarthrit.2024.152545

6. Shelmerdine SC, Di Paolo PL, Rieter J, Malattia C, Tanturri de Horatio L, Rosendahl K. A novel radiographic scoring system for growth abnormalities and structural change in children with juvenile idiopathic arthritis of the hip. Pediatr Radiol. 2018;48(8):1086-95. https://doi.org/10.1007/s00247-018-4136-6
7. Bertamino M, Rossi F, Pistorio A, Lucigrai G, Valle M, Viola S, Magni-Manzoni S, Malattia C, Martini A, Ravelli A. Development and initial validation of a radiographic scoring system for the hip in juvenile idiopathic arthritis. J Rheumatol. 2010;37(2):432-9. https://doi.org/10.3899/jrheum.090691
8. Tanturri de Horatio L, Shelmerdine SC, d'Angelo P, Di Paolo PL, Magni-Manzoni S, Malattia C, Damasio MB, Toma P, Avenarius D, Rosendahl K. A novel magnetic resonance imaging scoring system for active and chronic changes in children and adolescents with juvenile idiopathic arthritis of the hip. Pediatr Radiol. 2023;53(3):426-37. https://doi.org/10.1007/s00247-022-05502-8
9. Weiss PF, Maksymowych WP, Lambert RG, Jaremko JL, Biko DM, Paschke J, Brandon TG, Xiao R, Chauvin NA. Feasibility and reliability of the Spondyloarthritis Research Consortium of Canada sacroiliac joint inflammation score in children. Arthritis Res Ther. 2018;20(1):56. https://doi.org/10.1186/s13075-018-1543-x
10. Panwar J, Tse SML, Lim L, Tolend MA, Radhakrishnan S, Salman M, Moineddin R, Doria AS, Stimec J. Spondyloarthritis Research Consortium of Canada Scoring System for Sacroiliitis in Juvenile Spondyloarthritis/Enthesitis-related Arthritis: A Reliability, Validity, and Responsiveness Study. J Rheumatol. 2019;46(6):636-44. https://doi.org/10.3899/jrheum.180222
11. Ravelli A, Ioseliani M, Norambuena X, Sato J, Pistorio A, Rossi F, Ruperto N, Magni-Manzoni S, Ullmann N, Martini A. Adapted versions of the Sharp/van der Heijde score are reliable and valid for assessment of radiographic progression in juvenile idiopathic arthritis. Arthritis Rheum. 2007;56(9):3087-95. https://doi.org/10.1002/art.22835
12. Poznanski AK, Hernandez RJ, Guire KE, Bereza UL, Garn SM. Carpal length in children--a useful measurement in the diagnosis of rheumatoid arthritis and some concenital malformation syndromes. Radiology. 1978;129(3):661-8. https://doi.org/10.1148/129.3.661
13. Malattia C, Damasio MB, Pistorio A, Ioseliani M, Vilca I, Valle M, Ruperto N, Viola S, Buoncompagni A, Magnano GM, Ravelli A, Toma P, Martini A. Development and preliminary validation of a paediatric-targeted MRI scoring system for the assessment of disease activity and damage in juvenile idiopathic arthritis. Ann Rheum Dis. 2011;70(3):440-6. https://doi.org/10.1136/ard.2009.126862
14. Damasio MB, Malattia C, Tanturri de Horatio L, Mattiuz C, Pistorio A, Bracaglia C, Barbuti D, Boavida P, Juhan KL, Ording LS, Rosendahl K, Martini A, Magnano G, Toma P. MRI of the wrist in juvenile idiopathic arthritis: proposal of a paediatric synovitis score by a consensus of an international working group. Results of a multicentre reliability study. Pediatr Radiol. 2012;42(9):1047-55. https://doi.org/10.1007/s00247-012-2392-4
15. Angenete OW, Augdal TA, Rygg M, Rosendahl K. MRI in the Assessment of TMJ-Arthritis in Children with JIA; Repeatability of a Newly Devised Scoring System. Acad Radiol. 2022;29(9):1362-77. https://doi.org/10.1016/j.acra.2021.09.024
16. Augdal TA, Angenete OW, Shi XQ, Sall M, Fischer JM, Nordal E, Rosendahl K. Cone beam computed tomography in the assessment of TMJ deformity in children with JIA: repeatability of a novel scoring system. BMC Oral Health. 2023;23(1):12. https://doi.org/10.1186/s12903-022-02701-5
